# Supplementary material for: Prognostic Value of Measurable Residual Disease in Patients with AML Undergoing HSCT: A Multicenter Study
Source: Cancers (Basel). 2023 Mar 5;15(5):1609. doi: 10.3390/cancers15051609 (PMC10000405; doi:10.3390/cancers15051609)
Supplement: Supplementary file 1 [file cancers-15-01609-s001.zip › cancers-2135329-supplementary tables.pdf]

Supplementary Table S1. The information per fluorchrome from QA program.

	LAB_1	LAB_3	LAB_4
**2015**			
**Round I**			**25/33**<br>*CD4 PacB x3*<br>*CD5 PerCP5-5 x2*<br>*CD81 APCH7 x3*
**Round II**	**33/ 33**		**30/33**<br>*CD4 PacB x2*<br>*CD81 APCH7 x1*
**2016**			
**Round I**	**33/33**	**33/33**	**30/33**<br>*IgK PE x3*
**Round II**	**33/33**	**32/33**<br>*CD3-APC*	**28/33**<br>*IgK PE x3*<br>*CD81 APCH7 x2*
**2017**			
**Round I**	**31/33**<br>*IgK PE*<br>*CD81 APCH7*	**32/33**<br>*CD4-PacB*	**29/33**<br>*CD4 PacB x1*<br>*CD81 APCH7 x3*
**Round II**	**33/33**		**25/33**<br>*CD45 PacO x2*<br>*CD56-PE x1*<br>*IgK PE x2*<br>*CD81 APCH7 x3*
**2018**			
**Round I**	**32/33**<br>*CD4 PacB*	**33/33**	**33/33**
**Round II**	**33/33**	**32/33**<br>*CD81-APCH7*	**32/33**<br>*CD4 PacB*
**2019**			
**Round I**	**33/33**	**33/33**	**33/33**
**Round II**	**32/33**<br>*CD4 PacB*		**33/33**

**2020**<br>**Round I**<br>**Cyto 1**<br><br><br>**Cyto 2**	 <br>**32/33**<br>*CD38-APC-H7*<br><br>**33/33**	 <br>**31/33**<br>*CD45 PacO*<br>*CD38 APCH7*	 <br>**32/33**<br>*CD5 PerCP5-5*
**Round II**<br>**Cyto 1**<br><br><br>**Cyto 2**	**31/33**<br>*CD3-APC*<br>*CD4-PacB*<br><br>**32/33**<br>*CD3-APC*	**29/33**<br>*CD4-PAcB x3*<br>*CD38-APC-H7*	**29/33**<br>*CD5 PerCP5-5 x3*<br>*CD3-APC x1*

Supplementary Table S2. Designed panels to LAIP analysis

Combinations	Backbone	Different markers
1	HLADR, CD45, CD34, CD117	CD15, CD13 or CD123, CD33, CD38
3		CD15, NG2, CD33, CD14
4		CD64, CD56, CD33, CD14
5		CD7, CD13, CD33, CD38
6		CD15, CD56, CD123, CD4
7		nuTdT, CD56, CD123, CD4
8		CD64, CD56, CD300e, CD14
9		CD15, CD33 or CD13, CD19, CD38
10		CD15, CD33 or CD13, CD7, CD38
11		CD36, CD13, CD11b, CD71
12		CD15, CD56, CD33, CD38
13		nuTdT, CD13, CD33, CD38
14		CD7, CD56, CD33, CD38
15		CD16, CD13, CD33, CD10
16		CD36, CD64, CD33, CD71
17		CD15, CD13, CD22, CD38

Supplementary Table S3. Comparative analysis of MRD samples in the 4 laboratories: The table represents the MRD value determined by each laboratory.

	**Lab 1**	**Lab 2**	**Lab 3**	**Lab 4**
% MRD 1	0,54	0,55	0,39	0,54
% MRD 2	0,024	0,03	0,04	0,022
% MRD 3	0,27	0,24	0,23	0,18
% MRD 4	0,028	0,02	0,03	0,023
% MRD 5	0,002	0,003	0,005	0,002
% MRD 6	0,004	0,005	0,01	0,005

MRD: measurable residual disease

Supplementary Table S4. Univariate analysis of CIR, NRM, OS and LFS

Variable	CIR HR (95% CI)	NRM HR (95% CI)	OS HR (95% CI)	LFS HR (95% CI)
Sex				
male	1.02 (0.67-1.55) p 0.927	1 (0.56-1.77) p 0.998	1.07 (0.73-1.57). p 0.728	1.02 (0.73-1.44). p 0.883
Age	1.02 (1-1.04). p 0.037	1.02 (0.99-1.05). p 0.101	1.03 (1.01-1.04). p 0.002	1.02 (1.01-1.03). p 0.008
Conditioning				
RIC	1.37 (0.9-2.1). p 0.147	1.29 (0.72-2.3). p 0.393	1.73 (1.18-2.53). p 0.005	1.43 (1.01-2.01). p 0.041
Donor				
Unrelated	0.97 (0.63-1.51). p 0.91	2.04 (1.06-3.95). p 0.033	1.31 (0.87-1.97). p 0.196	1.29 (0.89-1.85). p 0.171
Haploidentical	0.45 (0.19-1.03). p 0.06	2.73 (1.16-6.42). p 0.021	1.15 (0.63-2.13). p 0.654	0.99 (0.56-1.75). p 0.981
Depletion T				
Yes	0.86 (0.5-1.48). p 0.583	1.09 (0.53-2.27). p 0.812	0.96 (0.58-1.58). p 0.876	0.92 (0.59-1.43). p 0.707
ELN2011				
Intermediate	2.11 (0.87-5.09). p 0.095	0.97 (0.40-2.38). p 0.948	1.41 (0.71-2.8). p 0.319	1.61 (0.86-2.98). p 0.134
Adverse	4.88 (1.99-11.99). p 0.01	1.48 (0.56-3.90). p 0.429	3.26 (1.62-6.55). p 0.001	3.75 (1.98-7.09). p <0.001
MRD ≥0.1	2.29 (1.43-3.65). p 0.001	1.54 (0.78-3.06). p 0.214	2.51 (1.64-3.83). p 0.000	2.29 (1.55-3.38). p 0.000

OS: overall survival; LFS: leukemia free survival HR: Hazard ratio; CI confidence interval; Myelo: myeloablative; RIC: reduce intensity conditioning; MRD: measurable residual disease; GVHD: Graf versus Host disease.

Supplementary Table S5. OS and LFS according to multivariate time-dependent analysis (time-dependent variable GvHD):

Variable	*LFS*	*OS*
Age	HR 1.01 [95% CI 0.99-1.03] p 0.218	HR 1.01 [95% CI 0.99-1.03] p 0.152
Conditioning		
*Myelo/RIC	HR 1.38 [95% CI 0.91-2.09] p 0.130	HR 1.68 [95% CI 1.05-2.68] p 0.029
ELN_2011		
*Intermediate	HR 1.8 [95% CI 0.95-3.45] p 0.072	HR 1.56 [95% CI 0.76-3.19] p 0.222
*Adverse	HR 3.43 [95% CI 1.76-6.7] p< 0.001	HR 2.8 [95% CI 1.33-5.89] p 0.006
MRD ≥0.1 before transplantation	1.89 [95% CI 1.25-2.86] p 0.003	HR 1.98 [95% CI 1.26-3.11] p 0.003
GvHD	HR 0.81 [95% CI 0.52-1.27] p0.366	HR 0.88 95% CI 0.53-1.45] p 0.608

CIR: cumulative incidence of relapse; NRM: non-relapse mortality; OS: overall survival; LFS: leukemia free survival; HR: Hazard ratio; CI confidence interval; Myelo: myeloablative; RIC: reduce intensity conditioning; MRD: measurable residual disease; GvHD: Graf versus Host disease.

Supplementary Table S6. Evolution of patients who did not relapse with positive MRD prior to transplantation.

	Type	Status disease at transplantation	aGvHD	cGVHD	MRD after	Last follow-up	Cause
**1**	**AML NPM1**	**1st CR MRD 0.66%**	**Grade 2**	**NO**	**0,02%**	DEATH (6 m)	VOD
**2**	AML NOS	Aplasia 2.4% blasts	Grade 4	Severe	NO	DEATH (3 y 1 m)	Unknown
**3**	AML CEBPA	1st CR MRD 0.12%	NO	mild	NO	ALIVE (4 y 2 m)	
**4**	AML NPM1	1st CR MRD 0.51%	Grade 1	mild	NO	ALIVE (3y 10m)	
**5**	AML NOS (IDH2)	1st CR MRD 0.15%	Grade 2	-	-	DEATH (3m)	Pneumonia
**6**	AML NOS WT1	1st CR MRD 0.01%	Grade 2	mild	NO	ALIVE (2y 9 m)	
**7**	AML NOS ASXL1	1st CR MRD 0.21%	Grade 4	-	0.26%	DEATH (2m)	aGVHD
**8**	TRML	1st CR MRD 0.3%	Grade 1	NO	NO	ALIVE (2 y y 6 m)	
**9**	AML KMT2	1st CR MRD (4.27%)	Grade 2	NO	NO	ALIVE (2 y y 9 m)	
**10**	AML -7	Aplasia (3.1% blasts)	Grade 1	Mild	NO	ALIVE (2 y y 1 mes)	
**11**	AML MCR (FLT3 0,13%)	1st CR MRD (0.2%)			NO	ALIVE (1 año y 7 m)	
**12**	AML NOS	1st CR MRD (0.2%)	Grade 1		NO	DEATH (6 m)	Pneumonia
**13**	AML t(6;9)	1st CR con MRD (1.04%)	Grade 1	Mild	NO	ALIVE (1 año y 3 m)	
**14**	AML NOS WT1	1st CR con MRD (0.11%)	Grade 2		NO	ALIVE (1 año y 3 m)	
**15**	AML MCR FLT3	Active disease (10% blasts)			NO	ALIVE (1 año y 2 m)	
**16**	AML NOS +7	1st CR con MRD (0.08%)	Grade 1	-	NO	DEATH (7 m)	Acute abdomen
**17**	AML NPM1	1st CR con MRD (0.1%)	Grade 2		NO	ALIVE (10 m)	
**18**	AML MCR KMT2A	1st CR con MRD (0.02%)	Grade 2		NO	ALIVE (10 m)	
**19**	AML CEBPA	1st CR con MRD (0.7%)	Grade 2	-	NO	ALIVE (3 m)	
**20**	AML t(9;11)	2nd CR con MRD (0.5%)			-	ALIVE (2.6 m)	
**21**	AML CRMD	1st CR con MRD (2.2%)	Grade 1	Moderate	NO	DEATH (8 m)	Infection
**22**	AML NPM/FLT3	2nd CR (0.2%)			NO	ALIVE (5 y y 8 m)	
**23**	AML inv16	2nd CR (0.08%)	Grade 2		NO	ALIVE (5 y y 6 m)	
**24**	AML NOS	1st CR (0.01%)	Grade 1	Mild	NO	ALIVE (5 y)	

**25**	AML del(20)(q12)	1st CR MRD (0.4%)		Mild	NO	ALIVE (5y 6 m)	
**26**	AML MCR (-7)	1st CR MRD (0.6%)			3% IS withdrawal	DEATH (6 m)	Infection MRD elevated
**27**	AML MCR RUNX1	1st CR MRD (0.4%)	Grade 4			DEATH (2 m)	Infection
**28**	AML IDH2	1st CR MRD (0.01%)	Grade 1	Severe	5% IS withdrawal	ALIVE (2 y)	
**29**	AML MCR t(16;17)	1st CR MRD (0.03%)	-	-	-	DEATH (<1m)	Infection
**30**	AML IDH2	1st CR MRD (0.01%)				ALIVE (2y 7 m)	
**31**	AML FLT3/NPM	1st CR MRD (0.02%)	Grade 2	Moderate		ALIVE (1y 3m)	
**32**	AML IDH	2nd CR MRD (2.1%)				ALIVE (9 m)	
**33**	AML RUNX1/RUNX1T(KIT mutation)	1st CR MRD (0.69%)	Grade 2			DEATH (3 m)	Infection
**34**	AML CBFB/MYH11	1st CR MRD (0.04%)		Severe	NO	ALIVE (4y 3 m)	
**35**	AML CBFB/MYH11	1st CR MRD (0.3%)	yes	Severe (after ILD)	NO	DEATH (1y 11m)	GVHD
**36**	AML 47XX	1st CR MRD 0.05%	Grade 2	-	NO	DEATH (8 m)	Septic shock
**37**	AML MCR	1st CR MRD (0.22%)		Mild		DEATH (2y 5 m)	Ureteral neoplasm
**38**	AML del(7q)	1st CR MRD (1%)	Grade 2			ALIVE (2 y 5 m)	
**39**	AML CBFB/MYH11	1st CR MRD (0.3%)			NO	ALIVE (2 y 4 m)	
**40**	AML NPM1	2nd CR MRD (0.2%)	Grade 4	Severe	0.2%	ALIVE (2y 2 m)	
**41**	AML NPM1	2nd CR MRD (0.01%)			Yes	ALIVE (4y 5m)	
**42**	AML t(7;13)	CR MRD (0.04%)	Grade 2	Extensive	-	ALIVE (1y 2 m)	
**43**	AML NOS	CR MRD (0.01%)	Grade 1	Extensive	-	ALIVE (2 y)	
**44**	AML NOS	CR MRD (0.16%)		Extensive	-	ALIVE (4 y)	
**45**	AML 47,XX, del(9)	CR MRD (0.13%)	Grade 2		-	ALIVE (4 y)	
**46**	AML 46XX	CR MRD (0.12%)		Extensive	-	ALIVE (5y 2 m)	

AML: acute myeloid leukemia; NOS: Not otherwise specified; CR: complete remission; MRD: measurable residual disease; GvHD: Graf versus Host disease.
