# Supplementary figures and images for: Prognostic Value of Measurable Residual Disease in Patients with AML Undergoing HSCT: A Multicenter Study
Source: Cancers (Basel). 2023 Mar 5;15(5):1609. doi: 10.3390/cancers15051609 (PMC10000405; doi:10.3390/cancers15051609)

Relapse  
after 1st CR

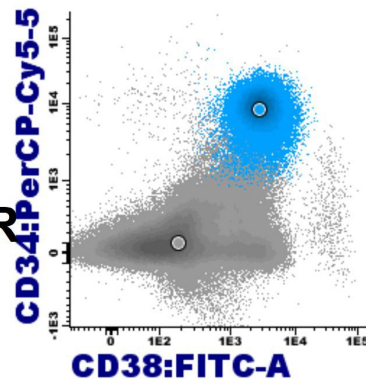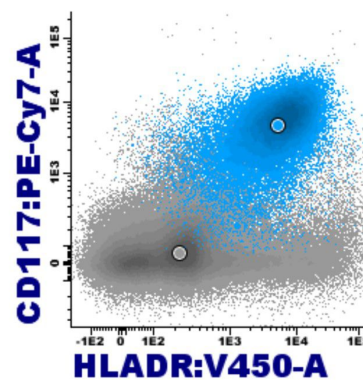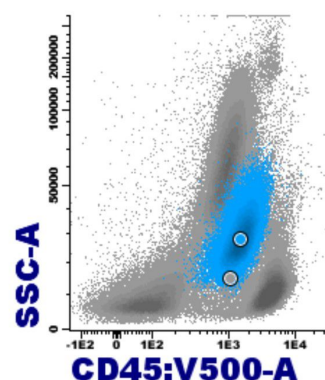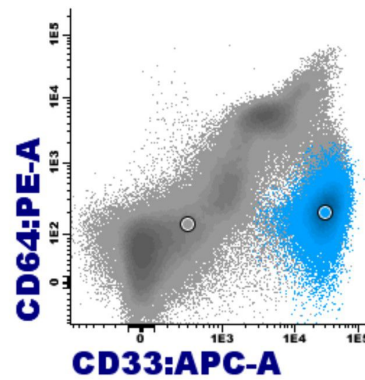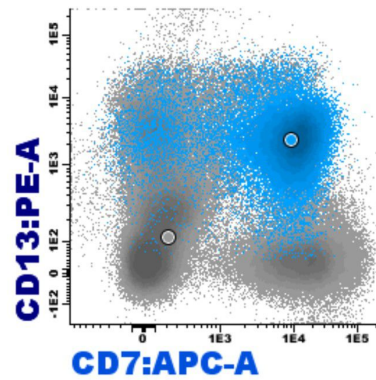

MRD  
Before  
HSCT

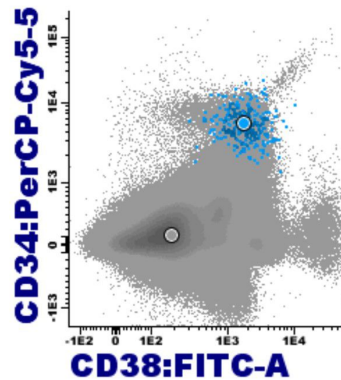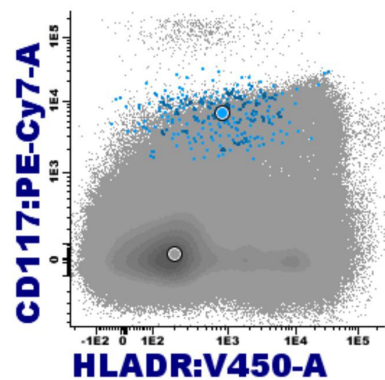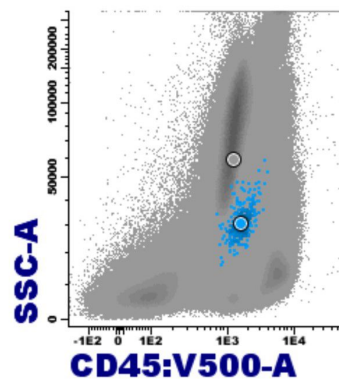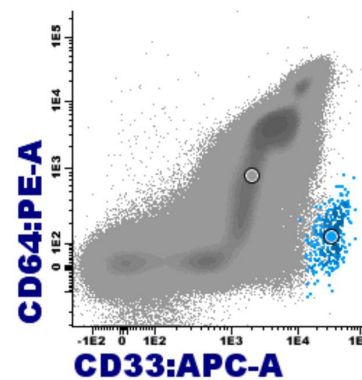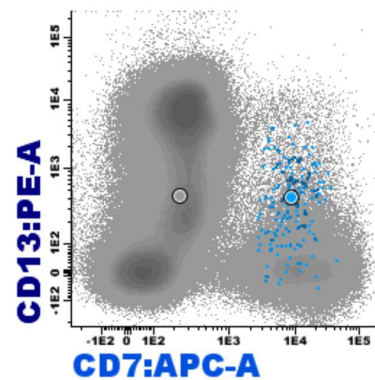

Supplement: Supplementary file 1 [file cancers-15-01609-s001.zip › Supplementary Figure S1.pdf]

## OS

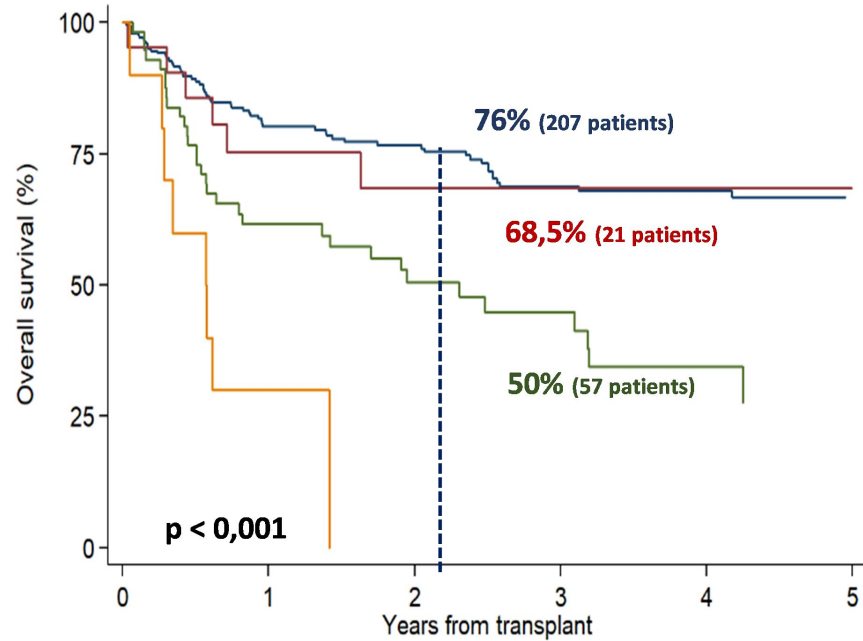

| Global         | 295 | 201 | 148 | 102 | 76 | 43 |
|----------------|-----|-----|-----|-----|----|----|
| MRD negative   | 207 | 155 | 120 | 85  | 65 | 38 |
| MRD < 0,1%     | 21  | 13  | 8   | 5   | 4  | 2  |
| MRD ≥ 0,1% <5% | 57  | 32  | 22  | 14  | 10 | 5  |
| Active disease | 10  | 1   |     |     |    |    |

## LFS

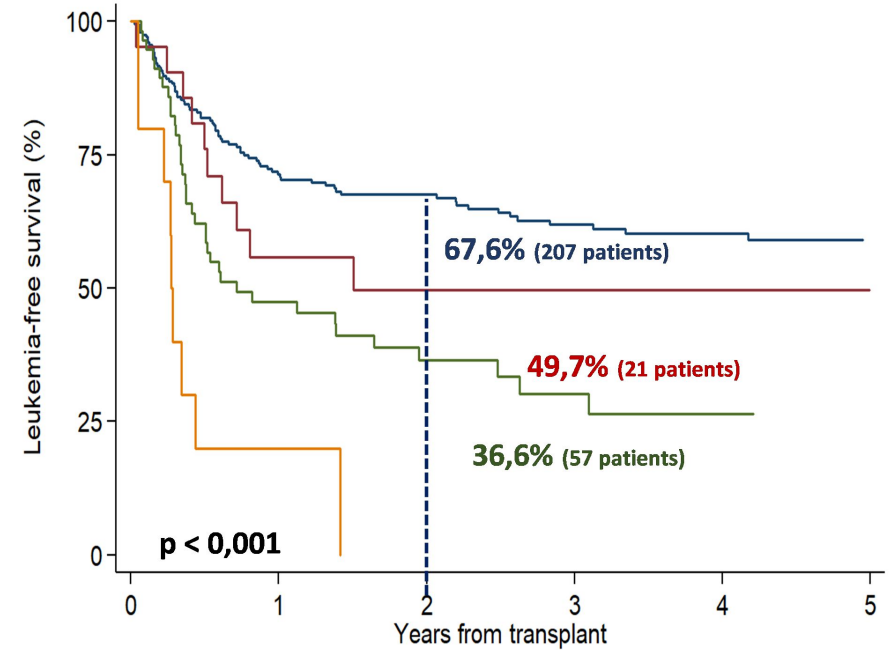

|                | 295 | 176 | 129 | 88 | 66 | 38 |
|----------------|-----|-----|-----|----|----|----|
| Global         | 207 | 140 | 107 | 76 | 58 | 34 |
| MRD negative   | 21  | 11  | 7   | 5  | 4  | 2  |
| MRD < 0,1%     | 57  | 25  | 17  | 9  | 7  | 4  |
| Active disease | 10  | 1   |     |    |    |    |

Supplement: Supplementary file 1 [file cancers-15-01609-s001.zip › Supplementary figure S2_OS.pdf]
